# Probing the anticancer mechanism of prospective herbal drug Withaferin A on mammals: a case study on human and bovine proteasomes

**DOI:** 10.1186/1471-2164-11-S4-S15

**Published:** 2010-12-02

**Authors:** Abhinav Grover, Ashutosh Shandilya, Virendra S Bisaria, Durai Sundar

**Affiliations:** 1 Department of Biochemical Engineering and Biotechnology, Indian Institute of Technology (IIT) Delhi, Hauz Khas, New Delhi 110016, India; 2 Supercomputing Facility for Bioinformatics and Computational Biology, Indian Institute of Technology (IIT) Delhi, Hauz Khas, New Delhi 110016, India

## Abstract

**Background:**

The UPP (ubiquitin proteasome pathway) is the major proteolytic system in the cytosol and nucleus of all eukaryotic cells which regulates cellular events, including mitotis, differentiation, signal transduction, apoptosis, and inflammation. UPP controls activation of the transcriptional factor NF-κB (nuclear factor κB), which is a regulatory protein playing central role in a variety of cellular processes including immune and inflammatory responses, apoptosis, and cellular proliferation. Since the primary interaction of proteasomes occurs with endogenous proteins, the signalling action of transcription factor NF-κB can be blocked by inhibition of proteasomes. A great variety of natural and synthetic chemical compounds classified as peptide aldehydes, peptide boronates, nonpeptide inhibitors, peptide vinyl sulfones and epoxyketones are now widely used as research tools for probing their potential to inhibit proteolytic activities of different proteasomes and to investigate the underlying inhibition mechanisms. The present work reports a bio-computational study carried out with the aim of exploring the proteasome inhibition capability of WA (withaferin A), a steroidal lactone, by understanding the binding mode of WA as a ligand into the mammalian proteasomes (X-ray crystal structure of *Bos taurus* 20S proteasome and multiple template homology modelled structure of 20S proteasome of *Homo sapiens*) using molecular docking and molecular dynamics simulation studies.

**Results:**

One possible mode of action which is proposed here for WA to act as a proteasome inhibitor is by suppression of the proteolytic activity which depends on the N-terminal threonine (Thr1) residue hydroxyl group. Docking studies carried out with herbal ligand WA into the structures of bovine and human proteasomes substantiate that WA has the ability to inhibit activity of mammalian 20S proteasomes by blocking the nucleophilic function of N-terminal Thr1. Results from molecular dynamics simulations in water show that the trajectories of both the native human 20S proteasome and the proteasome complexed with WA are stable over a considerably long time period of 4 ns suggesting the dynamic structural stability of human 20S proteasome/WA complex.

**Conclusions:**

Inhibition of proteasomal activity are promising ways to retard or block degradation of specific proteins to correct diverse pathologies. Though quite a number of selective and efficient proteasomal inhibitors exist nowadays, their toxic side effects limit their potential in possible disease treatment. Thus there is an indispensable need for exploration of novel natural products as antitumor drug candidates. The present work supports the mammalian proteasomes inhibiting activity of WA along with elucidation of its possible mode of action. Since WA is a small herbal molecule, it is expected to provide one of the modest modes of inhibition along with added favours of ease in oral administration and decreased immunogenicity. The molecular docking results suggest that WA can inhibit the mammalian proteasomes irreversibly and with a high rate through acylation of the N-terminal Thr1 of the β-5 subunit.

## Background

Ubiquitin is a small 76 amino acid protein conserved in all eukaryotic cells with a molecular weight of 8.6 kDa. When polyubiquitin is attached to target proteins, tagged proteins are selected for destruction by cytoplasmic organelles called proteasomes [1]. The mammalian 20S proteasome is characterized by a cylindrical shaped quaternary structure consisting of four heptameric stacked rings, α7β7β7α7, with 7 distinct α-type and 7 distinct β -type subunits, and with C2 symmetry similar to those of the yeast [2]. Within this multienzymatic proteasome system, a proteolytic multifunctional complex 26S proteasome is involved, which consists of a 19S regulatory particle and a 20S core particle [3,4]. The two outer α rings complex with the two 19S regulatory particles, forming a narrow channel through which only denatured proteins can pass [5]. The catalytic chamber is formed by the two inner rings, each of which contains three well-characterized proteolytic activities. In particular, three active subunits β1, β2, and β5 are responsible for the three major peptidase activities: the peptidylglutamil-hydrolase like, trypsin-like and chymotrypsin-like (ChT-L) activities respectively, as investigated by mutational and crystallographic studies [6,7]. The core particle is responsible for degradation of the proteins in a progressive manner, generating peptides of 3-25 amino acids in length [8].

The enzymatic activity of β-subunits is associated with the N-terminal threonine residues, which act as nucleophiles in the hydrolysis reaction catalyzing the cleavage of peptides through nucleophilic attack. Thus these proteasomes are classified as members of the Ntn (N-terminal nucleophilic) hydrolases group [1,9]. In the eukaryotic proteasome, out of the seven different β-subunit precursors which are processed during particle maturation by autolysis, only β1, β2 and β5 subunits can be activated by the autolytic process, with the release of the amino-terminal Thr1 functioning as the nucleophile [10-12].

UPP is the major proteolytic system in the cytosol and nucleus of all eukaryotic cells [13,14] which regulates cellular events including mitotis, differentiation, signal transduction, apoptosis, and inflammation [15]. UPP controls activation of the transcriptional factor NF-κB (nuclear factor κB), which is a regulatory protein playing central role in a variety of cellular processes, including immune and inflammatory responses, apoptosis, and cellular proliferation. Since the primary interaction of proteasomes occurs with endogenous proteins, the signalling action of transcription factor NF-κB can be blocked by inhibition of proteasomes, thus inhibiting the completion of the cell cycle and mitotic proliferation of cancerous cells and ultimately leading to cell death. It has been suggested that proteasomal activity is essential for tumour cell proliferation and development of drug resistance. Therefore, the development of specific inhibitors of proteasome mediated degradation pathway is now of considerable interest in the drug discovery research for cancer therapy and prevention [16]. A great variety of natural and synthetic chemical compounds classified as peptide aldehydes, peptide boronates, nonpeptide inhibitors, peptide vinyl sulfones, and epoxyketones are now widely used as research tools for studying their ability to inhibit proteolytic activity of various proteasomes from diverse origins.

Currently two proteasome inhibitors, bortezomib and NPI-0052, have been stated in clinical trials [17,18]. However, undesirable side effects such as fatigue, nausea, vomiting, peripheral neuropathy, anaemia, diarrhoea, and constipation have also been reported for these drugs [19]. Therefore, there has been an intensive drive to develop new proteasome inhibitors especially those of natural origin having little or no side effects.

**Figure 1:**
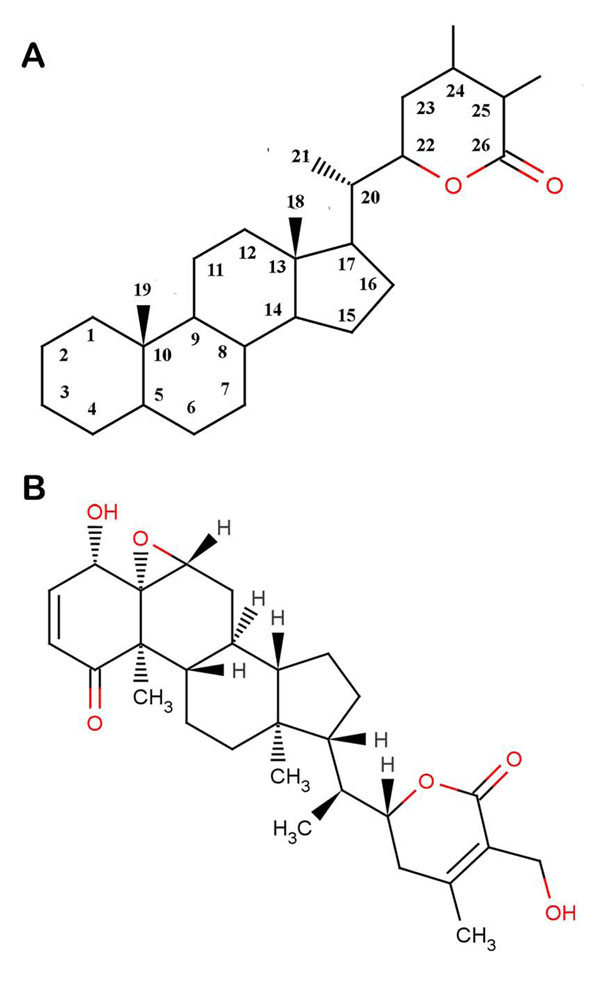
**Structures of withanolides.** (A) WA falls under the family of compounds known as withanolides which are a group of naturally occurring C28- steroidal lactones built on an intact or rearranged ergostane framework, in which C-22 and C- 26 are appropriately oxidized to form a six-membered lactone ring. The basic skeleton shown here is designated as the withanolide skeleton defined as a 22-hydroxyergostan-26-oic acid-26,22-lactone. (B) Structure of WA. It contains two sites which are prone to nucleophilic attack: A six membered δ-valero lactone ring containing a carbocyclic ester group and a three membered epoxy ring.

Naturally occurring inhibitors fall mainly in three groups being α’β’-epoxyketones, β-lactones and TMC-95s. WA, a principle constituent of the plant *Withania somnifera*, has received much attention in recent years owing to its various pharmacological properties like anti-inflammatory [20], antitumor [21], antibacterial [22], antioxidant [23], anticonvulsive [24,25] and immunosuppressive properties [26]. Most recently, it was shown to potentiate apoptosis of tumor cells by suppression of NF-κB activation [27-29]. Targeting of UPP has been identified as one of the mechanisms of WA activity exerting two distinct pharmacological activities; anti-tumor and anti-inflammatory [30]. Since proteasomes are required for nuclear translocation of p65/NF-κB which in turn results in activation of NF-κB, it is worth substantial to consider proteasomes as the target of WA. WA belongs to a family of steroidal lactones having withanolide skeleton as their basic structure (Figure 1A). It has been reported that C_1_ and C_24_ of WA are highly susceptible towards a nucleophilic attack [31]. As is evident from the structure of WA (Figure 1B) that it contains a lactone ring enclosed ester group, two conjugated ketone bonds and a three membered epoxy ring, all of which are quite susceptible to a nucleophilic attack. It has been hypothesized that WA can be a potent proteasome inhibitor and the mode of its action can be irreversible covalent modification. There is an evidence rationalising the proteasome inhibitory action of WA in which WA is shown to inhibit chymotrypsin like activity of a purified rabbit 20S proteasome (IC_50_=4.5µM) and 26S proteasome in human prostrate cancer cultures (at 5-10µM) and in xenografts (4-8 mg/Kg/day) [31]. Herein we report the ability of naturally occurring drug candidate WA and its mode of action for binding to mammalian 20S proteasomes as macromolecular receptors using computational approaches by analyzing the interactions between WA and the proteasomes.

## Methods

### Comparative protein structure multiple template homology modelling of Human 20S proteasome

The amino acid sequence of the β-5 subunit of h20S (human 20S proteasome) (GenBank: AAH57840.1) comprising of 263 amino acid residues was retrieved from NCBI. Since the crystal structure of h20S is not available, we used Modeller9v7 [32] to perform the homology modelling. The best possible templates were obtained using the build_profile python script. We built three dimensional structure of h20S using multiple template comparative homology modelling based on the following X-ray crystal structures obtained from PDB (Protein Data Bank)[33]: b20S (bovine 20S proteasome) at 2.75 Å resolution (PDB:1IRU), yeast 20S proteasome at 2.4 Å resolution (PDB:1RYP) and 20S proteasome from *Archaeoglobus fulgidus* (PDB:1J2Q). These template proteins were chosen based on a significant sequence similarity of h20S with these proteins in addition to their satisfactory crystallographic resolution. The alignment files of the targets and templates were then prepared using the align2D_mult python script (Sequence alignment module in MODELLER). These alignment files along with the x-ray crystal structures of the templates were used to generate the three dimensional structure models using the model_mult python script of MODELLER.

### Energy minimization

Discovery Studio (Version 1.7, Accelrys Software Inc.) was then used to execute energy minimization and to perform stereochemical quality checks to arrive at the best possible three dimensional structure of the protein. The force field applied was CHARMm and the energy minimization algorithm used was Conjugate Gradient with an RMS gradient of 0.1 using a maximum of 2000 steps. This resulted in model structures with considerably favourable potential energies. Furthermore, the variability among the models was used to evaluate the reliability of the modelling. The qualities of these models were analyzed by PROCHECKv3.4 [34].

### Binding pocket analysis

Binding Site analysis module of Discovery Studio was used to identify the putative binding pockets and protein ligand binding sites in the energy minimized three-dimensional structures of b20S and h20S.

### Ligand Docking

The energy minimized crystal structure of *Bos taurus* b20S (PDB: 1IRU) and the modelled structure of h20S were used to carry out molecular dockings. For ease in dockings, the subunits which are not directly interacting with the β-5 domain of the protein were removed of b20s crystal structure, and only the domains K, L, M, X & Y were retained for further analysis. The ligand molecule Withaferin-A [PubChem:265237] was retrieved from NCBI-PubChem Compound database [35].

AutoDock 4.0 suite was used as molecular-docking tool in order to carry out the docking simulations [36]. AutoDock 4.0 was launched in a Cygwin interface in the Windows operating system. Docking logs were analyzed in the graphical user interface of ADT (Auto dock Tools) [37]. Water molecules were cleaned off from the protein crystal structure before docking. H-atoms were added to these target proteins for correct ionization and tautomeric states of amino acid residues and the non-polar hydrogens were then merged up. Kollman united atom charges and solvation parameters were assigned to the proteins. Gasteiger charge was assigned to the ligand and then nonpolar hydrogens were merged. Rigid roots were also assigned to the ligand and five bonds were made “active” or rotatable. The modified structures so obtained: x-ray crystal structure of bovine, modelled three dimensional structure of human 20S proteasome, and the structure of ligand WA accounting the flexibility of its bonds, were converted to PDBQT format in ADT, as required in AutoDock calculations. The Lamarckian Genetic Algorithm was used with a population size of 150 dockings. Five million energy evaluations were used in the docking experiments. All other parameters, e.g. crossover rate and mutation rate, were run with default settings. The grid size for specifying the search space was set at 40 × 30 × 30 centered on Thr1 of the β5 subunit with a default grid point spacing of 0.375 Å. Energy scoring function of AutoDock 4 is based upon the calculation of pair-wise atomic terms including evaluations for different secondary interactions, dispersion/repulsion, hydrogen bonding, electrostatics, and desolvation [38]. Pre-calculated grid maps, which store grids of interaction energy based on the interaction of the ligand atom probes with receptor target, were obtained using AutoGrid. The user defined three dimensional grid must surround the region of interest in the macromolecule, and the ligand was limited to this search space during docking. The results are clustered into bins of similar conformations according to the cluster root mean square deviation (rmsd) and orientation.

### Confirmation of the docking results

The docking results obtained using AutoDock were also confirmed using ParDOCK [39] , which is an all atom energy based monte carlo docking protocol. Docking using ParDOCK requires a reference complex (target protein bound to a reference ligand) and a candidate molecule along with specific mention of the centre of mass of the cavity on which the ligand is to be docked.

### Molecular Dynamics simulations of human proteasome in water

The AMBER v.10 package [40] was used to prepare the protein and the ligand files as well as for the MD (Molecular Dynamics) simulations. The binding complex of h20S/WA obtained using ParDOCK and the free protein simulated in this study were neutralized by adding appropriate number of chloride counterions and were solvated in a octahedron box of TIP4P water with a 10 Å distance between the protein surface and the box boundary [41]. The partial atomic charges for the ligand were obtained using “antechamber” [42] module of Amber. The energy minimization and MD simulations of h20S and its complex with WA were carried out with the aid of the PMEMD module of the AMBER 10 program. First of all, the simulated binding complex was effected with a 2500 step minimization using the steepest descent algorithm followed by a 1000 step minimization using conjugate gradient to remove bad steric contacts. Topology and parameter files for the protein were generated using “ff03” and for the drug using “gaff” based on the atom types of the force field model developed by Cornell et al [43]. Then the system was equilibrated beginning with the protein atom restrained simulations having 150 ps equilibration dynamics of the solvent molecules at 300 K and a harmonic potential with a 10 kcal/mol restraint force. Next step involved the equilibration of the solute molecules with a fixed configuration of the solvent molecules in which the system was slowly heated from T = 10 to 300 K in 58 small intervals of 2.5 ps each for a total period of 145 ps. The entire system was then equilibrated at 300 K for 100 ps before a sufficiently long MD simulation (4 ns) at room temperature. The MD simulations were performed with a periodic boundary condition in the NPT ensemble at T=298.15 K with Berendsen temperature coupling [44] and constant pressure P=1 atm with isotropic molecule-based scaling . The SHAKE algorithm [45] was applied to fix all covalent bonds containing hydrogen atoms. We used a time step of 2 fs and a nonbond-interaction cut-off radius of 10 A°. The Particle Mesh Ewald (PME) method [46] was used to treat long-range electrostatic interactions. The coordinates of the trajectory was sampled every 1 ps for analysis of the energy stabilization and RMSD values of the protein as well as that of the complex. MD simulations were performed on a 320 processors SUN Microsystems clusters at Supercomputing Facility (SCFBio) at Indian Institute of Technology Delhi.

## Results and discussion

### Docking of WA into b20S proteasome

**Figure 2:**
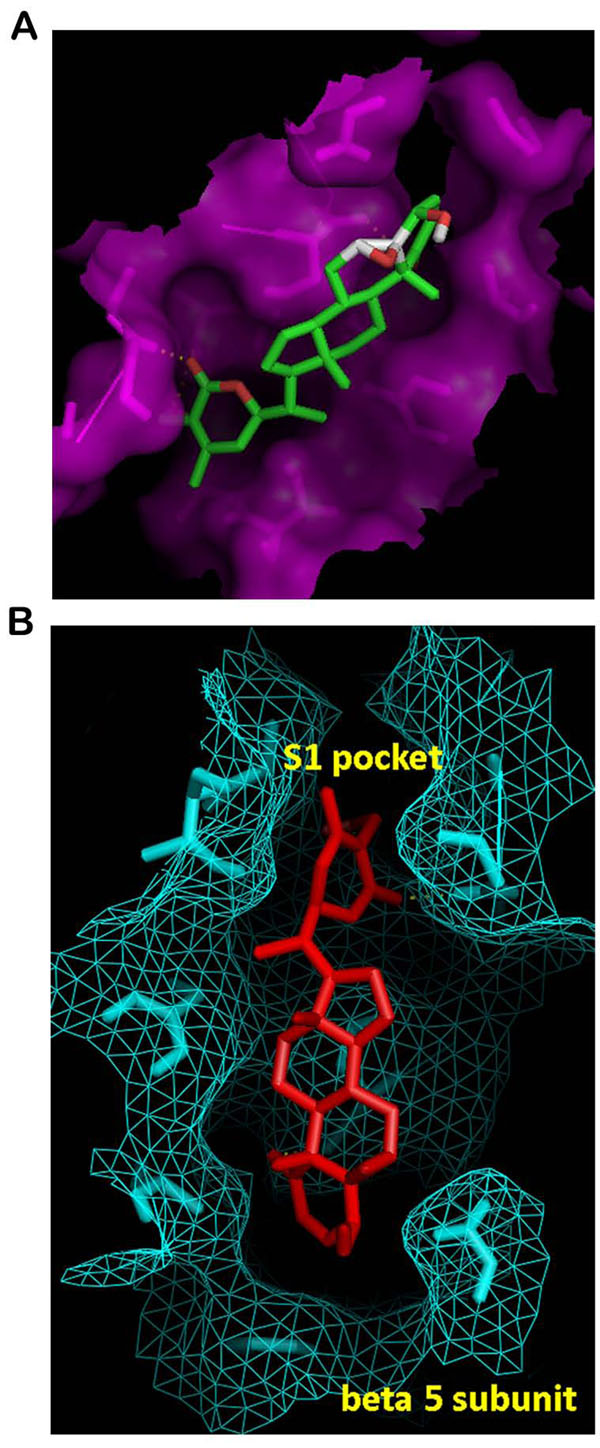
**Docking representations of WA into b20S.** (A) Docking of WA into the cavity of b20S. (B) Docked ligand being trapped inside the S1 pocket of the β-5 subunit of receptor mesh.

**Figure 3:**
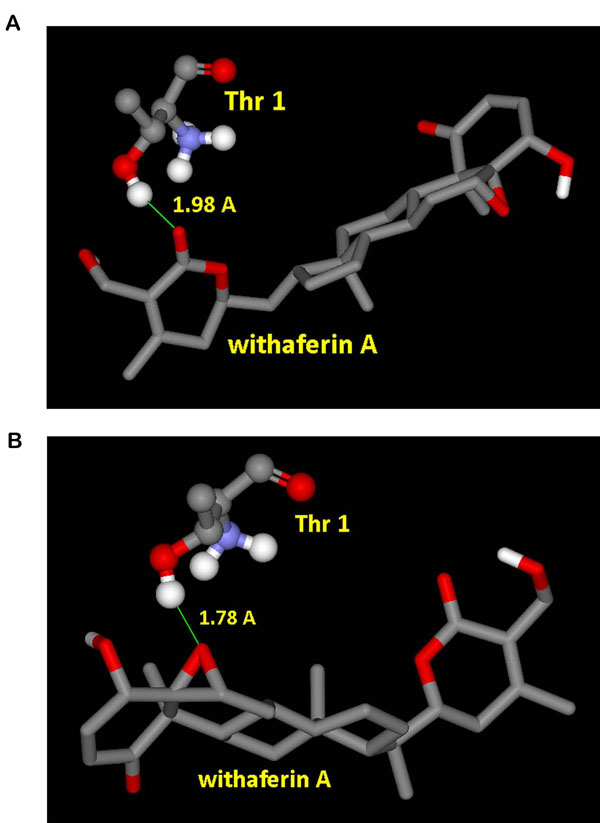
**Interactions of the ligand in different docked conformations of b20S.** (A) Docked conformation showing proximity of lactone ring’s carbonyl group of WA to the hydroxyl group of N-terminal Thr1 of β-5 subunit of b20S proteasome. (B) Docked conformation showing proximity of three membered epoxy ring of WA to the hydroxyl group of N-terminal Thr1 of β-5 subunit of b20S proteasome.

One possible mode of action which is proposed here for WA to act as a proteasome inhibitor is by suppression of the proteolytic activity which depends on the N-terminal threonine (Thr1) residue hydroxyl group, which is responsible for catalyzing the cleavage of peptides through nucleophilic attack. Using binding pocket analysis, S1 pocket of β-5 subunit was obtained as one of the putative binding site. As evident from the docking of WA into b20S (Figure 2A), WA is trapped inside this protein pocket. Figure 2B shows the ligand occupying the S1 cavity of the receptor being represented as a mesh surface. As AutoDock reports the best docking solution for each GA run and also performs a cluster analysis in which the total number of clusters and the rank of each docking mode (cluster rank) is reported, in 7 out of 10 docked conformations obtained by the clustering analysis at 2.0 Å, the carbonyl group of the lactone ring is found closest to the hydroxyl group of Thr1 (Figure 3A). The various properties of the docked conformation are shown in Table 1. The binding energies of the conformations of this cluster range from -6.37 to -6.11 Kcal/mol. The highest binding energy of -7.62 Kcal/mol was obtained for a conformation in which the epoxy group of the ligand is close to the nucleophilic hydroxyl group of the protein but with only a 10% clustering frequency (Figure 3B).

**Table 1:** Properties of the docked conformations

Receptor	b20S	b20S	h20S
Thr1 proximity to	Ester group of lactone ring	Epoxy ring	Ester group of lactone ring
**Binding Energy**	-6.37 Kcal/mol	-7.62 Kcal/mol	-6.92 Kcal/mol
**Ligand efficiency**	-0.19	-0.22	-0.2
**Inhibitoin constant**	21.29 µM	2.59 µM	8.45 µM
**Intermolecular energy**	-7.71 Kcal/mol	-8.07 Kcal/mol	-7.57 Kcal/mol
**Total internal energy**	-0.03 Kcal/mol	-0.93 Kcal/mol	-0.73 Kcal/mol

### Homology modelling of human 20S proteasome

The three dimensional structure of human proteasome was determined by comparative homology modelling with satisfaction of spacial constraints using multiple known X-ray crystal structures as templates. The chosen templates showed significant similarities to the h20S with e-values equal to 0. Five modelled three dimensional structures of human proteasome were obtained using Modeller out of which the model having the least DOPE score (Table 2) was chosen for the purpose of studying ligand and protein interactions. The quality and reliability of the model was ensured by assessing the backbone and side-chain conformations, bond lengths, angles, and residue contacts of the model through ProCheck, magnitudes of which are well within the criteria established for reliable structures (data not shown). The model was almost as good quality as those of the reference templates as evident from the results obtained using Ramachandran plot analysis (data not shown) for comparison of stereochemical and energetic properties of the models with those of the templates. The first 59 amino acids of the protein were then removed off from the structure as these are a part of a propeptide which is absent in the mature form. This model was energy minimized with an energy lowering of around 19,000 Kcal/mol and this energy minimized structure was used further for docking analysis.

**Table 2:** DOPE scores of the homology modelled structures

Model No.	DOPE Score
1	-24266.906
2	-24125.959
3	-24320.133
4	-24123.34
5	-23791.557

### Docking of WA into modelled h20S proteasome

**Figure 4:**
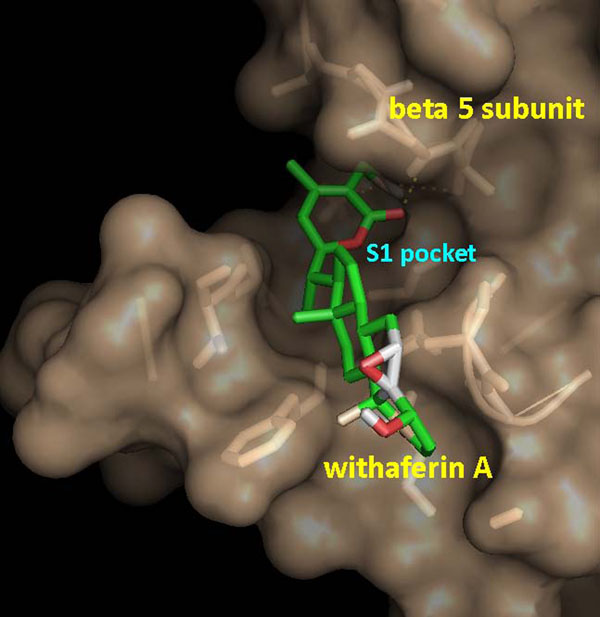
**Conformation of docked ligand occupying S1 pocket of the modelled h20S**.

**Figure 5:**
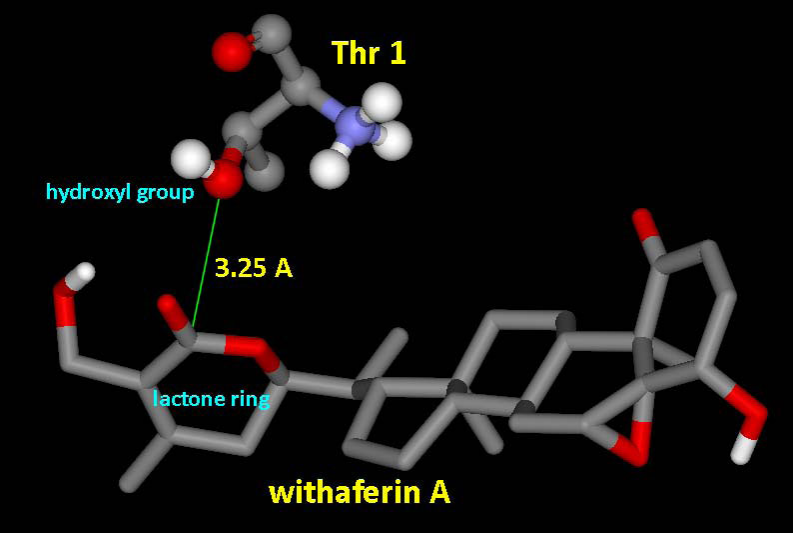
**Positioning of WA in the docked structure of h20S.** Lactone ring of WA positions itself quite close to the hydroxyl group of Thr1 of h20S receptor thus making itself prone to the nucleophilic attack by Thr1 of β-5 subunit.

Binding energy of -6.92 Kcal/mol was obtained from docking of WA into homology modelled h20S. The various properties listed in Table 1 provide sufficient results in order to support the ongoing mechanism of inhibition of h20S. Docked withefrin A positions itself into the S1 pocket of the receptor as shown in Figure 4. Moreover the ligand occupies the same conformation as required to facilitate the nucleophilic attack, positioning its lactone ring in vicinity of the hydroxyl nucleophile (Figure 5), with a clustering frequency of 30%.

It has been shown using kinetic analysis and X-ray diffraction studies that the ester bond of specific proteasome inhibitor lactacystin covalently modifies the N-terminal threonine of the β5-subunit, which is critical for proteasome inhibition [1,47-49]. Thus it is quite probable that lactacystin-like reaction occurs with WA also as it contains an internal ester bond in a δ-valero lactone ring (Figure 1B). A similar kind of cleavage of the lactone ring by serine protease has been reported in which the 3-benzyl-2-oxetanone, a β-lactone has been found to be a slowly hydrolyzed substrate of α-chymotrypsin [50]. Other herbal ligands like polyphenols [51] especially green tea polyphenols like EGCG and its analogs, genistein etc. [52,53], which have an ester bond susceptible to nucleophilic attack by Thr1 have also been reported to possess proteasome inhibition activity. Our results obtained from docking of WA into bovine and human 20S proteasome structures substantiate the proposed inhibition mechanism.

### MD simulations in water

**Figure 6:**
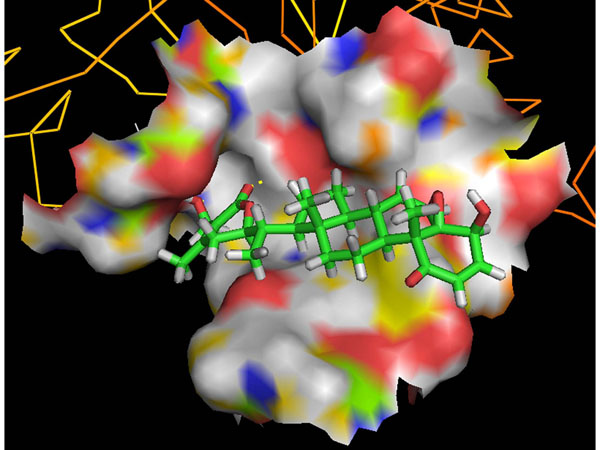
Docking representation of the drug WA inside the cavity of h20S obtained using ParDOCK.

**Figure 7:**
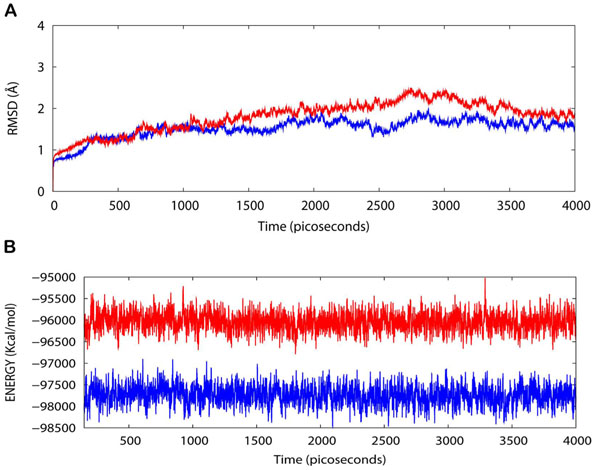
**(A) Plot of root mean square deviation (RMSD) of Cα of h20S (protein) and h20S/WA (complex).** RMSDs were calculated using the initial structures as templates. For protein (red) the reference is the modelled structure and for complex (blue) the reference is the initial model. The trajectories were captured every 1 ps until the simulation time reached 4000 ps. **(B) Plot of total energy of h20S and h20S/WA (complex).** The energy trajectories of both the protein (red) and the complex (blue) are stable over the entire length of simulation time.

The h20S/WA protein-drug binding complex with the binding energy of -6.91 kcal/mol obtained using ParDOCK (Figure 6) was used for carrying out MD simulations. After MD simulations, we calculated RMSDs between Cα trajectory of h20S and Cα of its modelled structure recorded every 1 ps. The RMSDs for the trajectory of h20S complexed with WA were also calculated using its initial model as a reference structure. The results in Figure 7A show that the RMSDs of the trajectory of the complex were always less than 2 Å for the entire simulation suggesting the stability of our simulation system. The trajectories were not greatly different from the modelled structure, with only minor movements of the Cα of the protein observed. The adherence of the total energy trajectories to more or less constant values for both the complex and the protein were seen during the entire simulation length (Figure 7B), with the energy values of the complex much lowered than that of the native protein indicating thermodynamic stability of the complex. The simulation length used in this study was long enough to allow rearrangement of side chains of the native as well as the drug complexed protein to find their most stable binding mode. Thus the present MD simulations along with the molecular docking experiments made clear the dynamic structural stability of h20S in complex with the drug WA, together with the inhibitory mechanism.

## Conclusions

Since proteasomes play an essential role in the turnover of cellular proteins, modulation or inhibition of proteasomal activity are thus promising ways to retard or block degradation of specific proteins in order to correct diverse pathologies. Though nowadays there exist quite a number of selective and efficient proteasomal inhibitors, the toxic side effects of these compounds strongly limit their potential in possible disease treatment. Thus there is an indispensable need for exploration of novel natural products as anti-cancer drug candidates. The study conducted here makes use of molecular docking and molecular dynamics simulation approaches, which include the search in space for the energetically most favorable conformation of a protein-ligand complex and the scoring of the resulting geometries with respect to binding energy, to analyze the proteasome inhibitory potential of WA and to investigate the underlying inhibitory mechanism. We have obtained significant results delineating the mammalian proteasomes’ inhibitory activity of WA alongwith elucidation of its possible mode of action. Since WA is a small herbal molecule, it is expected to provide one of the modest modes of inhibition alogwith added favors of ease in oral administration and decreased immunogenicity. Conclusively it is strongly suggested here that WA is a potent proteasome inhibitor and should be looked forward for further clinical investigations as a possible proteasome inhibitory drug candidate.

## Authors' contributions

AG, VSB and DS designed the methods and experimental setup. AG carried out the implementation of the various methods. AS assisted AG in this process. AG and DS wrote the manuscript. All authors have read and approved the final manuscript.

## Competing interests

The authors declare that they have no competing interests.

## References

[B1] Groll M Ditzel L Lowe J Stock D Bochtler M Bartunik HD Huber R Structure of 20S proteasome from yeast at 2.4 angstrom resolution Nature 1997 386 6624 463 471 10.1038/386463a0 9087403

[B2] Unno M Mizushima T Morimoto Y Tomisugi Y Tanaka K Yasuoka N Tsukihara T The structure of the mammalian 20S proteasome at 2.75 angstrom resolution Structure 2002 10 5 609 618 10.1016/S0969-2126(02)00748-7 12015144

[B3] Peters JM Proteasomes - Protein-Degradation Machines of the Cell Trends Biochem Sci 1994 19 9 377 382 10.1016/0968-0004(94)90115-5 7985232

[B4] Voges D Zwickl P Baumeister W The 26S proteasome: A molecular machine designed for controlled proteolysis Annu Rev Biochem 1999 68 1015 1068 10.1146/annurev.biochem.68.1.1015 10872471

[B5] Peters JM Franke WW Kleinschmidt JA Distinct 19-S and 20-S Subcomplexes of the 26-S Proteasome and Their Distribution in the Nucleus and the Cytoplasm J Biol Chem 1994 269 10 7709 7718 8125997

[B6] Groll M Heinemeyer W Jager S Ullrich T Bochtler M Wolf DH Huber R The catalytic sites of 20S proteasomes and their role in subunit maturation: A mutational and crystallographic study P Natl Acad Sci USA 1999 96 20 10976 10983 10.1073/pnas.96.20.10976 PMC34229 10500111

[B7] Heinemeyer W Fischer M Krimmer T Stachon U Wolf DH The active sites of the eukaryotic 20 S proteasome and their involvement in subunit precursor processing J Biol Chem 1997 272 40 25200 25209 10.1074/jbc.272.40.25200 9312134

[B8] Nussbaum AK Dick TP Keilholz W Schirle M Stevanovic S Dietz K Heinemeyer W Groll M Wolf DH Huber R Cleavage motifs of the yeast 20S proteasome beta subunits deduced from digests of enolase 1 P Natl Acad Sci USA 1998 95 21 12504 12509 10.1073/pnas.95.21.12504 PMC22860 9770515

[B9] Brannigan JA Dodson G Duggleby HJ Moody PCE Smith JL Tomchick DR Murzin AG A Protein Catalytic Framework with an N-Terminal Nucleophile Is Capable of Self-Activation Nature 1995 378 6555 416 419 10.1038/378416a0 7477383

[B10] Chen P Hochstrasser M Autocatalytic subunit processing couples active site formation in the 20S proteasome to completion of assembly Cell 1996 86 6 961 972 10.1016/S0092-8674(00)80171-3 8808631

[B11] Schmidtke G Kraft R Kostka S Henklein P Frommel C Lowe J Huber R Kloetzel PM Schmidt M Analysis of mammalian 20S proteasome biogenesis: The maturation of beta-subunits is an ordered two-step mechanism involving autocatalysis Embo J 1996 15 24 6887 6898 9003765 PMC452515

[B12] Seemuller E Lupas A Baumeister W Autocatalytic processing of the 20S proteasome Nature 1996 382 6590 468 470 10.1038/382468a0 8684489

[B13] Lupas A Zwickl P Wenzel T Seemuller E Baumeister W Structure and function of the 20S proteasome and of its regulatory complexes Cold Spring Harb Sym 1995 60 515 524 10.1101/sqb.1995.060.01.055 8824424

[B14] Hershko A Ciechanover A The ubiquitin system Annu Rev Biochem 1998 67 425 479 10.1146/annurev.biochem.67.1.425 9759494

[B15] Myung J Kim KB Crews CM The ubiquitin-proteasome pathway and proteasome inhibitors Med Res Rev 2001 21 4 245 273 10.1002/med.1009 11410931 PMC2556558

[B16] Borissenko L Groll M 20S proteasome and its inhibitors: crystallographic knowledge for drug development Chem Rev 2007 107 3 687 717 10.1021/cr0502504 17316053

[B17] Adams J The development of proteasome inhibitors as anticancer drugs Cancer Cell 2004 5 5 417 421 10.1016/S1535-6108(04)00120-5 15144949

[B18] Ruiz S Krupnik Y Keating M Chandra J Palladino M McConkey D The proteasome inhibitor NPI-0052 is a more effective inducer of apoptosis than bortezomib in lymphocytes from patients with chronic lymphocytic leukemia Mol Cancer Ther 2006 5 7 1836 1843 10.1158/1535-7163.MCT-06-0066 16891470

[B19] Colson K Doss DS Swift R Tariman J Thomas TE Bortezomib, a newly approved proteasome inhibitor for the treatment of multiple myeloma: nursing implications Clin J Oncol Nurs 2004 8 5 473 480 10.1188/04.CJON.473-480 15515281

[B20] Alhindawi MK Alkhafaji SH Abdulnabi MH Antigranuloma Activity of Iraqi Withania-Somnifera J Ethnopharmacol 1992 37 2 113 116 10.1016/0378-8741(92)90069-4 1434685

[B21] Mishra L Singh B Dagenias S Scientific basis for the therapeutic use of Withania somnifera (ashwagandha): a review Altern Med Rev 2000 5 334 336 10956379

[B22] Owais M Sharad KS Shehbaz A Saleemuddin M Antibacterial efficacy of Withania somnifera (ashwagandha) an indigenous medicinal plant against experimental murine salmonellosis Phytomedicine 2005 12 3 229 235 10.1016/j.phymed.2003.07.012 15830846

[B23] Bhattacharya A Ghosal S Bhattacharya SK Anti-oxidant effect of Withania somnifera glycowithanolides in chronic footshock stress-induced perturbations of oxidative free radical scavenging enzymes and lipid peroxidation in rat frontal cortex and striatum J Ethnopharmacol 2001 7 4 1 6 10.1016/S0378-8741(00)00309-3 11137343

[B24] Kulkarni SK George B Mathur R Protective effect of Withania somnifera root extract on electrographic activity in a lithium-pilocarpine model of status epilepticus Phytotherapy Research 1998 12 6 451 453 10.1002/(SICI)1099-1573(199809)12:6<451::AID-PTR328>3.0.CO;2-C

[B25] Kulkarni S George B Mathur R Protective effect of Withania somnifera root extract on electrographic activity in a lithium pilocarpine model of status epilepticus Phytother Res 1998 12 451 453 10.1002/(SICI)1099-1573(199809)12:6<451::AID-PTR328>3.0.CO;2-C

[B26] Furmanowa M Gajdzis-Kuls D Ruszkowska J Czarnocki Z Obidoska G Sadowska A Rani R Upadhyay SN In vitro propagation of Withania somnifera and isolation of withanolides with immunosuppressive activity Planta Med 2001 67 2 146 149 10.1055/s-2001-11494 11301861

[B27] Kaileh M Vanden W Berghe Heyerick A Horion J Piette J Libert C De Keukeleire D Essawi T Haegeman G Withaferin A strongly elicits I kappa B kinase beta hyperphosphorylation concomitant with potent inhibition of its kinase activity J Biol Chem 2007 282 7 4253 4264 10.1074/jbc.M606728200 17150968

[B28] Oh JH Kwon TK Withaferin A inhibits tumor necrosis factor-alpha-induced expression of cell adhesion molecules by inactivation of Akt and NF-kappa B in human pulmonary epithelial cells Int Immunopharmacol 2009 9 5 614 619 10.1016/j.intimp.2009.02.002 19236958

[B29] Ichikawa H Takada Y Shishodia S Jayaprakasam B Nair MG Aggarwal BB Withanolides potentiate apoptosis, inhibit invasion, and abolish osteoclastogenesis through suppression of nuclear factor-kappa B (NF-kappa B) activation and NF-kappa B-regulated gene expression Mol Cancer Ther 2006 5 6 1434 1445 10.1158/1535-7163.MCT-06-0096 16818501

[B30] Mohan R Hammers H Bargagna-Mohan P Zhan X Herbstritt C Ruiz A Zhang L Hanson A Conner B Rougas J Withaferin A is a potent inhibitor of angiogenesis Angiogenesis 2004 7 2 115 122 10.1007/s10456-004-1026-3 15516832

[B31] Yang HJ Shi GQ Dou QP The tumor proteasome is a primary target for the natural anticancer compound withaferin a isolated from "Indian Winter Cherry" Mol Pharmacol 2007 71 2 426 437 10.1124/mol.106.030015 17093135

[B32] Sali A Blundell TL Comparative Protein Modeling by Satisfaction of Spatial Restraints J Mol Biol 1993 234 3 779 815 10.1006/jmbi.1993.1626 8254673

[B33] Berman HM Westbrook J Feng Z Gilliland G Bhat TN Weissig H Shindyalov IN Bourne PE The Protein Data Bank Nucleic Acids Res 2000 28 1 235 242 10.1093/nar/28.1.235 10592235 PMC102472

[B34] Kleywegt GJ Jones TA Phi/psi-chology: Ramachandran revisited Structure 1996 4 12 1395 1400 10.1016/S0969-2126(96)00147-5 8994966

[B35] NCBI-PubChem Compound database http://pubchem.ncbi.nlm.nih.gov/

[B36] Morris GM Goodsell DS Halliday RS Huey R Hart WE Belew RK Olson AJ Automated docking using a Lamarckian genetic algorithm and an empirical binding free energy function J Comput Chem 1998 19 614 1639 1662 10.1002/(SICI)1096-987X(19981115)19:14<1639::AID-JCC10>3.0.CO;2-B

[B37] Sanner MF Python: A programming language for software integration and development J Mol Graph Model 1999 17 1 57 61 10660911

[B38] Bikadi Z Hazai E Application of the PM6 semi-empirical method to modeling proteins enhances docking accuracy of AutoDock J Cheminform 2009 1 15 10.1186/1758-2946-1-15 20150996 PMC2820493

[B39] Gupta A Gandhimathi A Sharma P Jayaram B ParDOCK: An all atom energy based Monte Carlo docking protocol for protein-ligand complexes Protein Peptide Lett 2007 14 7 632 646 10.2174/092986607781483831 17897088

[B40] Case DA Darden TA Cheatham TE Simmerling CL Wang J Duke RE Luo R Crowley M Walker RC Zhang W AMBER 10 2008 University of California

[B41] Jorgensen WL Chandrasekhar J Madura JD Impey RW Klein ML Comparison of Simple Potential Functions for Simulating Liquid Water J Chem Phys 1983 70 2 926 935 10.1063/1.445869

[B42] Jakalian A Bush BL Jack DB Bayly CI Fast, efficient generation of high-quality atomic Charges. AM1-BCC model: I. Method J Comput Chem 2000 21 2 132 146 10.1002/(SICI)1096-987X(20000130)21:2<132::AID-JCC5>3.0.CO;2-P 12395429

[B43] Cornell WD Cieplak P Bayly CI Gould IR Merz KM Ferguson DM Spellmeyer DC Fox T Caldwell JW Kollman PA A second generation force field for the simulation of proteins, nucleic acids, and organic molecules J Am Chem Soc 1996 118 9 2309 2309 10.1021/ja955032e

[B44] Berendsen HJC Postma JPM Vangunsteren WF Dinola A Haak JR Molecular-Dynamics with Coupling to an External Bath J Chem Phys 1984 81 8 3684 3690 10.1063/1.448118

[B45] Ryckaert JP Ciccotti G Berendsen HJC Numerical-Integration of Cartesian Equations of Motion of a System with Constraints - Molecular-Dynamics of N-Alkanes J Comput Phys 1977 23 3 327 341 10.1016/0021-9991(77)90098-5

[B46] Essmann U Perera L Berkowitz ML Darden T Lee H Pedersen LG A Smooth Particle Mesh Ewald Method J Chem Phys 1995 103 19 8577 8593 10.1063/1.470117

[B47] Kim DH Park JI Chung SJ Park JD Park NK Han JH Cleavage of beta-lactone ring by serine protease. Mechanistic implications Bioorgan Med Chem 2002 10 8 2553 2560 10.1016/S0968-0896(02)00108-6 12057644

[B48] Dick LR Cruikshank AA Grenier L Melandri FD Nunes SL Stein RL Mechanistic studies on the inactivation of the proteasome by lactacystin A central role for clasto-lactacystin beta-lactone J Biol Chem 1996 271 13 7273 7276 10.1074/jbc.271.13.7273 8631740

[B49] Kisselev AF Goldberg AL Proteasome inhibitors: from research tools to drug candidates Chem Biol 2001 8 6590 739 758 10.1016/S1074-5521(01)00056-4 11514224

[B50] Laskowski RA Macarthur MW Moss DS Thornton JM Procheck - a Program to Check the Stereochemical Quality of Protein Structures J Appl Crystallogr 1993 26 283 291 10.1107/S0021889892009944

[B51] Mozzicafreddo M Cuccioloni M Cecarini V Eleuteri AM Angeletti M Homology Modeling and Docking Analysis of the Interaction between Polyphenols and Mammalian 20S Proteasomes J Chem Inf Model 2009 49 2 401 409 10.1021/ci800235m 19434841

[B52] Smith DM Daniel KG Wang ZG Guida WC Chan TH Dou QP Docking studies and model development of tea polyphenol proteasome inhibitors: Applications to rational drug design Proteins 2004 54 1 58 70 10.1002/prot.10504 14705024

[B53] Dou QP Landis-Piwowar KR Chen D Huo C Wan SB Chan TH Green tea polyphenols as a natural tumour cell proteasome inhibitor Inflammopharmacology 2008 16 5 208 212 10.1007/s10787-008-8017-8 18815743 PMC3303149

